# A Rare Case of Immunoglobulin A Vasculitis in an Adult Male

**DOI:** 10.7759/cureus.56422

**Published:** 2024-03-18

**Authors:** Prakash Shende, Avani Reddy, Ahsan A Faruqi, Tejas Kore

**Affiliations:** 1 General Medicine, Dr. D. Y. Patil Medical College, Hospital and Research Centre, Pune, IND

**Keywords:** glucocorticoid, proteinuria, palpable purpura, malena, iga vasculitis (igav)

## Abstract

A 40-year-old Indian male presented with rash and abdominal pain, leading to a diagnosis of IgA vasculitis, a rare condition in adults. This systemic vasculitis involves IgA immune complex deposition, resulting in inflammation and tissue damage. Diagnosis relies on clinical features and biopsy findings, with management focused on symptom relief and addressing organ involvement. Long-term prognosis varies, emphasizing the importance of multidisciplinary care and patient education for optimal outcomes.

## Introduction

Henoch-Schönlein purpura (HSP), now known as immunoglobulin A vasculitis (IgAV), is a kind of systemic vasculitis that is characterized by immunological deposits that are predominantly IgA and impact small blood vessels. Palpable purpura, arthralgia, dark urine, and acute kidney injury are common symptoms. IgAV is uncommon in adults but more common in children, accounting for up to 45% of pediatric vasculitides. IgA vasculitis tends to progress more severely in adults, requiring more intensive treatment strategies [1].

IgAV affects adults at a rate of 0.8-2.2 cases per 100,000 people annually with a male-to-female ratio of 1.5 [2]. It has been noted that IgAV is associated with obesity, diabetes, and hypertension. Purpura, arthralgia, and abdominal discomfort make up the "classic triad" of IgAV [2].

## Case presentation

A 40-year-old male presented to us with a rash on both upper and lower limbs for two weeks, intermittent abdominal pain, and two episodes of black-colored stools.

He gave a history of rash starting over both lower limbs, insidious in onset, and progressing to both upper limbs. The rash was reddish purple in color, with well-defined, non-itchy patches. The rash was also associated with diffuse joint pains. He had no history of fever, decreased urine output, blood in the urine, drug consumption, exposure to heavy metals, or breathlessness. Abdominal pain was described as two episodes per week of intermittent spasmodic pain, not localized, diffuse in nature which would subside within a few minutes, with no aggravating or relieving factors, not associated with vomiting. He also gave a history of black-colored stools, two to three episodes in two weeks, not associated with hard/loose stools, no abdominal pain during the passage of stools, not associated with any fresh blood in stools or any other bleeding tendencies. The patient had a prior history of a common cold two weeks prior to the onset of the rash. He had no known comorbidities.

**Figure 1:**
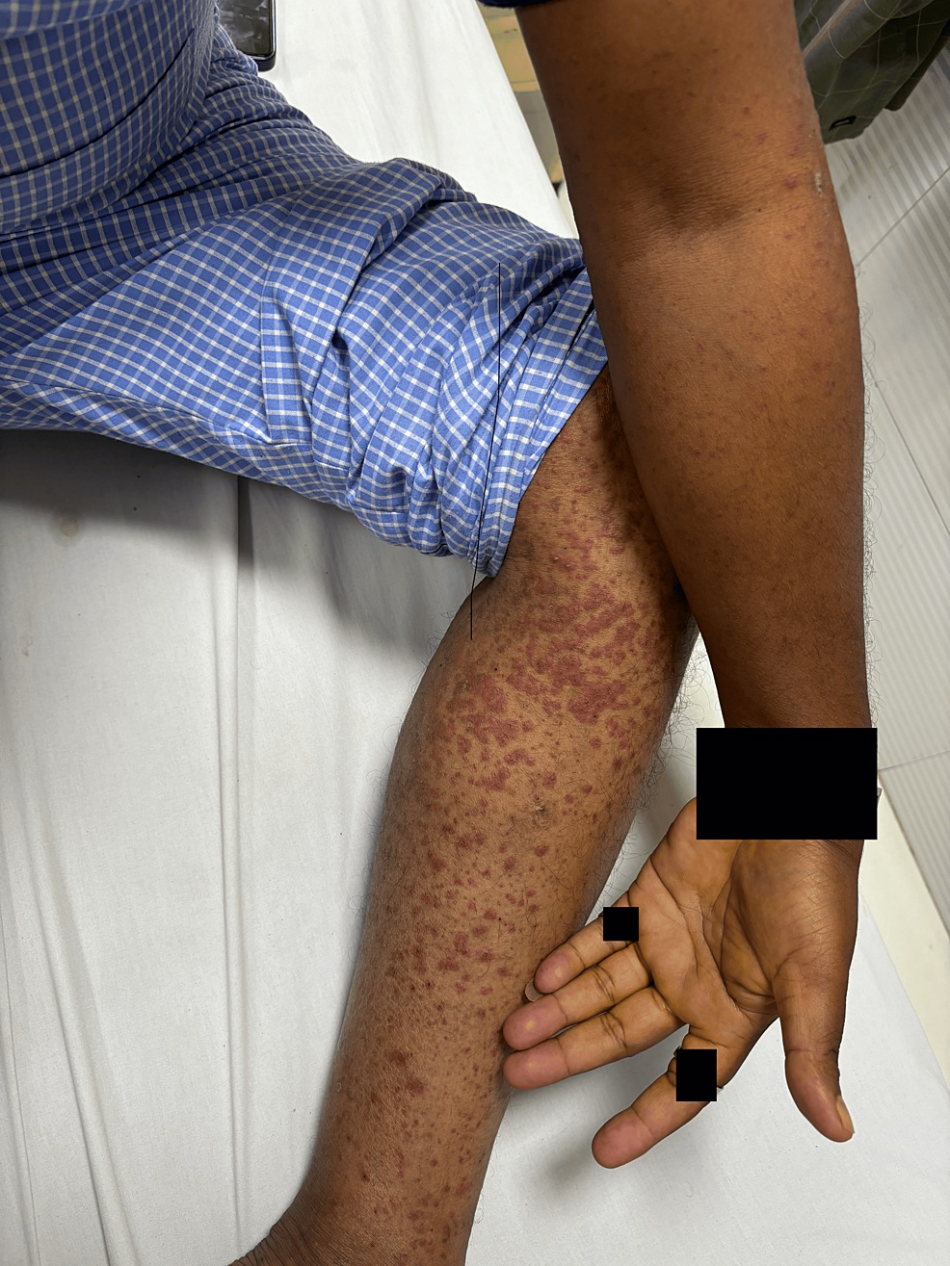
Photograph showing purpuric rash

On examination, blood pressure was 170/100mmHg, pulse rate was 88/min, oxygen saturation was 98% on room air, and the random blood sugar level was 285mg/dl. Local examination of both upper and lower limbs showed reddish purple, well-defined palpable purpura, spreading all over both legs, buttocks, and upper limbs, sparing the abdomen. No lymphadenopathy was noted. Systemic examination revealed no abnormalities. Chest X-ray and electrocardiography were within normal limits. Ultrasonography of the abdomen and pelvis revealed Grade I fatty liver. Blood investigations were sent to further evaluate the patient (Tables 1, 2). Purpuric rash was seen on presentation as shown in Figure 1.

**Table 1:** Blood reports on admission SGOT: Serum glutamic oxaloacetic transaminase; SGPT: Serum glutamic pyruvic transaminase; ALP: Alkaline phosphate; TSH: Thyroid stimulating hormone; CRP: C-reactive protein; ESR: Erythrocyte sedimentation rate; Ig E: Immunoglobulin E; HbA1c: Glycated hemoglobin, HIV: Human immunodeficiency viruses; HbsAg: Hepatitis B surface antigen; HCV-Ab: Hepatitis C virus antibodies

Blood tests	Result	Reference range
Hemoglobin	15.30g/dl	12.3-15.3g/dl
Total leucocyte count	10,000/ul	4000-10000/ul
Platelets	390,000/ul	150000-400000/ul
Eosinophils	6%	1-6%
Absolute neutrophils	8160	2000-7000/ul
Serum urea	19mg/dl	17-49mg/dl
Serum creatinine	0.87mg/dl	0.6-1.2mg/dl
Serum total bilirubin	0.52mg/dl	0.10-1.20mg/dl
Conjugated bilirubin	0.19mg/dl	0.2-0.3mg/dl
Unconjugated bilirubin	0.33mg/dl	0.1-1.0mg/dl
SGOT	38U/Lt	8-43U/Lt
SGPT	23U/Lt	7-55U/Lt
ALP	102U/Lt	35-104U/Lt
Total protein	7.4g/dl	6.0-8.3g/dl
Serum albumin	3.8g/dl	3.4-5.0g/dl
Serum globulin	3.5g/dl	2.3-3.5g/dl
Serum sodium	138mmol/Lt	136-145mmol/Lt
Serum potassium	3.76mmol/Lt	3.50-5.10mmol/Lt
Serum chloride	105mmol/Lt	98-107mmol/Lt
HbA1C	7.80%	4.0-5.6%
Total cholesterol	192mg/dl	<200mg/dl
Triglycerides	148mg/dl	148mg/dl
TSH	1.37uIU/L	0.35-4.94uIU/L
CRP	25mg/L	5mg/L
ESR	51mm/hr	Up to 15mm/hr
Serum IgE	160U/ml	150-300IU/ml
Urine routine microscopy	Protein - trace glucose-1+	Within normal limit
HIV	Non-reactive	Non-reactive/reactive
HbsAg	Non-reactive	Non-reactive/reactive
HCV-Ab	Non-reactive	Non-reactive/reactive

**Table 2:** Blood test results on days three and four of admission ANA blot: Antinuclear antibody blot test; ANCA: Antinuclear cytoplasmic antibodies; PR3: Proteinase 3; MPO: Myeloperoxidase

Blood tests	Result	Reference range
Serum IgA	610mg/dl	60 to 356mg/dl
ANCA - MPO (p-ANCA)	Negative (0.806)	Negative <20RU/ml, positive >20RU/ml
ANCA - PR3 (c-ANCA)	Negative (<2)	Negative <20RU/ml, positive >20RU/ml
Rheumatoid factor	Negative (10)	Negative: Below 14.00IU/ml
ANA blot	Negative	Negative/positive for antinuclear antibodies
24-hour urine protein	1399mg/24hr	<149mg/24hr
Stool for occult blood	Negative	Negative/positive

In other investigations that were done, a contrast-enhanced CT scan of the abdomen and pelvis showed only changes in cystitis and borderline hepatomegaly. Upper GI Endoscopy showed a medium, oval-shaped ulcer with irregular margins and covered with a whitish slough noted in the proximal body - Forrest’s class III, patchy erythema in fundus, body, and antrum.

For the skin biopsy a 4mm punch biopsy was taken from his right leg. The report showed a diagnosis of leukocytoclastic vasculitis.

His blood sugar levels and blood pressure were monitored routinely and we labeled him as newly diagnosed with type 2 diabetes mellitus with hypertension, he was treated for the same.

Considering all the investigations, blood and radiological, coupled with the skin biopsy we diagnosed the patient with IgA vasculitis, suspected to be triggered by his prior common cold infection. The patient was treated with oral prednisolone 80mg/day, which was tapered off gradually in follow-up visits. Reduction of purpura almost entirely and absence of proteinuria was noted in follow-up as well, showing good response to glucocorticoid therapy.

## Discussion

IgA is activated by external stimuli, particularly microorganisms, it is thought that a variety of microbes might cause atypical IgA autoimmune reactions in the host. It is accepted that a wide variety of microorganisms and viruses are involved in the pathophysiology of IgA vasculitis. *Streptococcus aureus*, *Helicobacter pylori*, varicella-zoster virus, hepatitis virus, Parvovirus, human immunodeficiency virus, cytomegalovirus, and *Clostridium difficile* are the representative causal pathogens. As a result, these microbes act as catalysts to cause IgA vasculitis [3]. As seen in our case, the patient had a history of a common cold, two weeks prior to the onset of the rash.

Abdominal symptoms are common in HSP, affecting up to 85% of patients due to bowel and mesentery inflammation [4]. The pain is colicky and hard to pinpoint, sometimes leading to mistaken diagnoses of acute abdomen. Children may show signs of intussusception. Gastrointestinal bleeding occurs in about one-third of cases. However, similar symptoms can be seen in other conditions, requiring careful differential diagnosis. Joint pain, especially in ankles, is symmetrical and may require steroid treatment. Renal involvement, signaled by proteinuria and hematuria, typically appears within the first three months of the disease [4].

Renal complications significantly impact the long-term health outcomes of patients with IgA vasculitis. Approximately 11% of patients with kidney involvement progress to end-stage renal disease (ESRD) [2]. Factors associated with poor prognosis include impaired renal function at the start, proteinuria exceeding 1g/day at disease onset, the presence of macroscopic hematuria, and hypertension. Over a 10-year period, up to 50% of patients with these risk factors may progress to ESRD [2].

Early treatment with oral prednisone is beneficial for the management of gastrointestinal, joint, and renal symptoms. Evidence suggests that prednisone reduces the length and severity of stomach discomfort during the first two weeks of treatment, based on many randomized control trials. Rituximab, azathioprine, mycophenolate mofetil, prednisone, and cyclophosphamide are recommended for second-line therapy [5].

Prednisone medication was compared to a placebo in research including 171 individuals who were monitored for six months. When compared to a placebo, prednisone, when administered at a dose of 1mg/kg/day for two weeks and then tapered gradually over the following two weeks, successfully decreased the intensity of joint and stomach discomfort. Prednisone was effective in treating renal problems, but it did not stop them from occurring. More specifically, compared to 34% of patients receiving a placebo, 61% of patients treated with prednisone saw a remission of renal symptoms [6].

In our case, early glucocorticoid treatment proved to be efficient in allowing symptomatic relief and preventing further complications as well.

## Conclusions

Given the range of complications that can arise from IgA vasculitis, including ESRD, all patients should have a comprehensive evaluation. A prompt diagnosis can enable glucocorticoid treatment, leading to a satisfactory remission of symptoms. Regardless of the patient's age or gender, the rare incidence described in this case report ought to be considered in our differential diagnosis when we encounter patients with similar symptoms and presentations.
